# Supplementary material for: Performance of joint modelling of time-to-event data with time-dependent predictors: an assessment based on transition to psychosis data
Source: PeerJ. 2016 Oct 19;4:e2582. doi: 10.7717/peerj.2582 (PMC5075698; doi:10.7717/peerj.2582)
Supplement: Supplemental Information 4 — The data was used to illustrate the various analytic methods discussed in the paper. The data include the subject numbers, family history of mental illness (yes/no), start day of a period, end day of a period, transition to psychosis status (yes/no) and depression severity score. [file peerj-04-2582-s004.pdf]

```
"subj","famhist","startday","endday","transtat","dept"
1,0,0,32,0,30
1,0,32,60,0,11
1,0,60,88,0,16
1,0,88,119,1,24
2,0,0,39,0,22
2,0,39,88,0,24
2,0,88,144,0,12
2,0,144,186,0,8
2,0,186,237,0,10
2,0,237,270,0,12
2,0,270,300,0,10
2,0,300,347,0,13
2,0,347,400,0,16
3,1,0,35,0,5
3,1,35,98,0,2
3,1,98,169,0,4
3,1,169,210,0,2
3,1,210,231,0,2
3,1,231,317,0,10
3,1,317,350,0,1
3,1,350,380,0,8
3,1,380,408,0,9
3,1,408,408.5,0,4
4,1,0,53,1,23
5,0,0,99,0,16
5,0,99,666,0,22
5,0,666,707,0,26
6,1,0,56,0,8
6,1,56,91,0,4
6,1,91,126,0,3
6,1,126,159,0,2
6,1,159,197,0,3
6,1,197,216,1,20
7,1,16,65,0,9
7,1,65,94,0,10
7,1,94,122,0,9
7,1,122,166,0,11
7,1,166,199,0,12
7,1,199,234,0,9
7,1,234,276,0,26
7,1,276,305,0,19
7,1,305,334,0,10
7,1,334,361,0,26
7,1,361,388,0,16
7,1,388,388.5,0,15
8,1,0,121,1,39
9,1,0,55,0,24
9,1,55,90,0,23
9,1,90,123,0,20
9,1,123,157,0,20
9,1,157,192,0,26
9,1,192,245,0,8
9,1,245,272,0,21
9,1,272,301,0,19
9,1,301,335,0,18
9,1,335,370,0,8
9,1,370,370.5,0,14
10,1,0,7,1,29
11,1,0,73,0,14
11,1,73,105,0,16
11,1,105,154,0,4
11,1,154,182,1,11
12,1,0,138,0,15
12,1,138,259,0,16
12,1,259,287,0,17
12,1,287,347,1,19
13,0,0,45,0,15
```

13,0,45,101,0,NA
13,0,101,129,0,14
13,0,129,168,0,17
13,0,168,209,0,7
13,0,209,259,0,9
13,0,259,292,0,16
13,0,292,327,0,23
13,0,327,362,0,22
13,0,362,404,0,21
13,0,404,404.5,0,11
14,1,0,600,0,22
14,1,600,600.5,0,8
15,0,31,108,1,NA
16,1,8,50,0,7
16,1,50,112,0,6
16,1,112,173,0,6
16,1,173,209,1,4
17,1,0,18,1,21
18,0,0,77,0,16
18,0,77,129,0,32
18,0,129,159,0,13
18,0,159,199,0,10
18,0,199,227,0,14
18,0,227,262,0,9
18,0,262,290,0,6
18,0,290,332,0,10
18,0,332,377,0,20
18,0,377,426,0,6
18,0,426,426.5,0,13
19,1,0,53,0,17
19,1,53,96,0,17
19,1,96,158,0,14
19,1,158,186,0,39
19,1,186,214,0,32
19,1,214,242,0,25
19,1,242,272,0,23
19,1,272,307,0,17
19,1,307,350,1,28
20,1,28,63,0,15
20,1,63,92,0,25
20,1,92,158,0,17
20,1,158,189,0,15
20,1,189,220,0,16
20,1,220,248,0,19
20,1,248,280,0,17
20,1,280,308,0,16
20,1,308,336,0,11
20,1,336,367,0,16
20,1,367,401,0,15
20,1,401,401.5,0,10
21,0,0,68,0,27
21,0,68,456,1,4
22,1,0,42,0,18
22,1,42,122,0,7
22,1,122,520,0,10
22,1,520,520.5,0,21
23,1,0,41,0,15
23,1,41,77,0,6
23,1,77,120,0,23
23,1,120,147,0,18
23,1,147,203,0,19
23,1,203,238,0,9
23,1,238,348,0,11
23,1,348,390,0,12
23,1,390,419,0,8
23,1,419,613,0,8
24,1,0,39,1,21
25,1,0,35,0,7

```
25,1,35,66,0,6
25,1,66,95,1,3
26,1,0,39,0,24
26,1,39,123,0,11
26,1,123,193,0,6
26,1,193,271,0,4
26,1,271,313,0,0
26,1,313,348,0,4
26,1,348,481,0,1
26,1,481,481.5,0,1
27,0,0,37,0,16
27,0,37,65,0,9
27,0,65,94,0,8
27,0,94,138,0,14
27,0,138,181,0,17
27,0,181,213,0,13
27,0,213,253,0,16
27,0,253,280,0,26
27,0,280,325,0,21
27,0,325,354,0,22
27,0,354,382,0,16
27,0,382,487,0,12
27,0,487,487.5,0,19
28,1,0,15,1,24
29,0,0,38,0,5
29,0,38,95,0,6
29,0,95,130,0,5
29,0,130,165,0,2
29,0,165,200,0,5
29,0,200,229,0,8
29,0,229,257,0,3
29,0,257,300,0,3
29,0,300,334,0,5
29,0,334,367,0,2
29,0,367,396,0,0
29,0,396,396.5,0,2
30,1,0,28,0,17
30,1,28,57,0,21
30,1,57,93,0,24
30,1,93,119,0,11
30,1,119,165,0,22
30,1,165,203,0,1
30,1,203,231,0,15
30,1,231,259,0,16
30,1,259,308,0,9
30,1,308,339,0,12
30,1,339,339.5,0,18
31,0,0,27,0,NA
31,0,27,55,0,11
31,0,55,104,0,16
31,0,104,176,0,10
31,0,176,209,0,17
31,0,209,244,0,12
31,0,244,272,0,15
31,0,272,310,0,14
31,0,310,359,0,11
31,0,359,406,0,7
31,0,406,581,0,12
32,1,0,28,0,24
32,1,28,52,0,21
32,1,52,108,0,16
32,1,108,150,0,19
32,1,150,192,0,9
32,1,192,224,0,13
32,1,224,259,0,15
32,1,259,294,0,17
32,1,294,329,0,26
32,1,329,357,0,21
```

32,1,357,392,0,16
32,1,392,608,0,14
33,0,0,35,0,13
33,0,35,64,0,5
33,0,64,91,0,9
33,0,91,119,0,13
33,0,119,147,0,7
33,0,147,231,0,13
33,0,231,260,0,17
33,0,260,294,0,15
33,0,294,336,0,5
33,0,336,412,0,4
33,0,412,413,0,6
34,0,0,463,0,27
34,0,463,463.5,0,15
35,0,0,49,0,2
35,0,49,84,0,0
35,0,84,119,0,2
35,0,119,147,0,4
35,0,147,182,0,1
35,0,182,225,0,1
35,0,225,274,0,6
35,0,274,351,0,6
35,0,351,392,0,2
35,0,392,392.5,0,0
36,0,0,28,0,22
36,0,28,67,0,10
36,0,67,315,0,15
36,0,315,386,0,9
36,0,386,423,0,25
36,0,423,456,0,9
36,0,456,742,1,6
37,1,0,116,0,11
37,1,116,217,0,9
37,1,217,251,0,1
37,1,251,294,0,4
37,1,294,329,0,6
37,1,329,369,0,7
37,1,369,369.5,0,7
38,1,0,307,0,14
38,1,307,378,0,11
38,1,378,398,0,6
39,0,0,32,0,19
39,0,32,57,0,10
39,0,57,94,0,6
39,0,94,123,0,12
39,0,123,164,0,20
39,0,164,185,0,20
39,0,185,234,0,11
39,0,234,282,0,7
39,0,282,336,0,12
39,0,336,372,0,8
39,0,372,372.5,0,6
40,1,0,40,0,19
40,1,40,56,1,23
41,0,0,28,0,20
41,0,28,70,0,12
41,0,70,110,0,12
41,0,110,172,0,3
41,0,172,214,0,3
41,0,214,252,0,0
41,0,252,411,0,0
41,0,411,411.5,0,4
42,0,0,35,0,9
42,0,35,71,0,9
42,0,71,99,0,4
42,0,99,133,0,6
42,0,133,168,0,6

```
42,0,168,217,0,8
42,0,217,269,1,25
43,1,0,30,1,39
44,1,0,30,0,7
44,1,30,59,0,6
44,1,59,85,0,4
44,1,85,119,0,8
44,1,119,147,0,7
44,1,147,176,0,0
44,1,176,217,0,11
44,1,217,253,0,8
44,1,253,296,0,9
44,1,296,329,0,3
44,1,329,359,0,1
44,1,359,420,0,1
44,1,420,420.5,0,1
45,0,0,28,0,16
45,0,28,62,0,14
45,0,62,95,0,13
45,0,95,118,1,19
46,0,0,66,0,14
46,0,66,90,0,8
46,0,90,114,0,2
46,0,114,146,0,9
46,0,146,182,0,3
46,0,182,204,0,6
46,0,204,240,0,4
46,0,240,267,0,3
46,0,267,316,0,1
46,0,316,359,0,4
46,0,359,399,0,12
46,0,399,497,0,4
47,0,0,29,0,19
47,0,29,64,1,10
48,1,0,23,0,10
48,1,23,56,0,7
48,1,56,87,0,8
48,1,87,128,0,5
48,1,128,165,0,15
48,1,165,199,0,17
48,1,199,235,0,13
48,1,235,298,0,3
48,1,298,333,0,10
48,1,333,378,0,22
48,1,378,424,0,22
48,1,424,424.5,0,7
49,1,0,29,1,25
```
